# Cognitive individual differences are key in the network of trait covariance

**DOI:** 10.1093/beheco/arag027

**Published:** 2026-03-02

**Authors:** Gaia De Russi, Elia Gatto, Mattia Lanzoni, Giuseppe Castaldelli, Cristiano Bertolucci, Tyrone Lucon-Xiccato

**Affiliations:** Department of Life Sciences and Biotechnology, University of Ferrara, Via Luigi Borsari 46, 44121 Ferrara, Italy; Department of Life Sciences and Biotechnology, University of Ferrara, Via Luigi Borsari 46, 44121 Ferrara, Italy; Department of Chemical, Pharmaceutical and Agricultural Sciences, University of Ferrara, Via Luigi Borsari 46, 44121 Ferrara, Italy; Department of Environmental and Prevention Sciences, University of Ferrara, Via Luigi Borsari 46, 44121 Ferrara, Italy; Department of Environmental and Prevention Sciences, University of Ferrara, Via Luigi Borsari 46, 44121 Ferrara, Italy; Department of Life Sciences and Biotechnology, University of Ferrara, Via Luigi Borsari 46, 44121 Ferrara, Italy; Department of Life Sciences and Biotechnology, University of Ferrara, Via Luigi Borsari 46, 44121 Ferrara, Italy

**Keywords:** cognitive ecology, conservation behavior, fish cognition, individual differences, personality

## Abstract

Individual differences in cognition are widespread across animal taxa, and despite progress in identifying possible mechanisms shaping evolution and maintenance of this variation, significant gaps in our understanding persist. A crucial but often overlooked factor is the covariation between cognitive traits and traits from other domains, which can impose evolutionary constraints and drive indirect selection, shaping cognitive variance. To explore this factor, the present study investigated the covariation between cognitive, behavioral, physiological, and life-history traits in a wild-caught fish, the tench (*Tinca tinca*). We focused on 5 cognitive traits: motor lateralization, visual lateralization, spatial learning, cognitive flexibility, and memory. These cognitive traits were interrelated, with for instance, faster learners exhibiting lower cognitive flexibility. Additionally, cognitive traits correlated with several behavioral and physiological traits. Using a network analysis, we demonstrated the relevance of cognitive variance in the relationship among traits. Visual lateralization was central in the network, connecting subnetworks including behavioral and physiological traits. Memory was strongly linked to physiological traits. Last, spatial learning, cognitive flexibility, and visual lateralization were the traits with the highest impact in the whole network. Overall, our study not only highlights the complex associations between cognitive, behavioral and physiological, but also underscores the critical role of cognition in these relationships. This finding supports the idea that the evolution and maintenance of cognitive variance are both influenced by, and exert remarkably influence on, the variation in other traits.

## Teaser

Animal cognitive abilities can vary widely among individuals. This study examined how individual differences in cognition covary with behavioral, physiological, and life-history traits. Results showed a stable covariation pattern. The majority of the significant covariations involved at least 1 cognitive trait. A network analysis highlighted a central position of cognitive traits in the structure of trait covariation. These findings provide insights into how cognitive differences can evolve and be maintained due to indirect selection and constraints from other traits.

## Introduction

Individual differences in cognition have been reported across all main vertebrate taxa (mammals: Thornton and Lukas 2012; Hanke et al. 2022; birds: Cussen 2017; Bobrowicz and Greiff 2022; teleost fish: Lucon-Xiccato and Bisazza 2017) and even in invertebrates (Lucon-Xiccato et al. 2024). From an evolutionary perspective, the emergence and maintenance of cognitive variance present an intricate puzzle because many studies have reported that enhanced cognition provides fitness advantages in nature (Huebner et al. 2018; Rochais et al. 2022; Fichtel et al. 2023). Therefore, one would expect the presence of directional selection acting on cognition, which should erode variance. The evidence of persisting cognitive variance suggests that factors other than directional selection may be at play.

Various hypotheses have been proposed to explain factors that may contribute to cognitive variance. In some cases, direct energetic tradeoffs involving brain tissues underlying cognition have been hypothesized (Aiello and Wheeler 1995; Heldstab et al. 2022) and demonstrated (Kotrschal et al. 2013), and they can probably contribute to individual differences in cognition. A more recent hypothesis has suggested that individual differences in cognitive abilities are related to individual differences in behavior (Carere and Locurto 2011). This, in part, may be a mechanistic consequence: in nature, individuals with different behaviors, such as varying levels of exploratory tendency and activity, may encounter stimuli at different rates, thereby affecting certain aspects of cognitive performance, such as learning rate (Sih and Del Giudice 2012). However, a relationship between behavioral and cognitive traits has also been documented in numerous controlled laboratory studies where exposure to stimuli is standardized (see references in Dougherty and Guillette 2018). Moreover, in some cases, this relationship is observed in directions opposite to those expected if it were merely a consequence of behavioral differences in the testing environment (eg, White et al. 2017). This suggests a more intrinsic association, in which behavioral phenotypes are inherently linked to cognitive traits. Such findings imply that natural selection may favor combinations where individuals with certain behavioral traits tend to express particular cognitive traits, or vice versa. In this context, considering behavioral traits alongside cognitive ones may provide deeper insight into the sources of cognitive variation. For example, frequency-dependent selection maintaining individual differences in behavior (Wolf and McNamara 2012; Lichtenstein and Pruitt 2015) could also generate indirect selection that maintains variation in cognition through the covariation between these 2 domains (discussed in Lucon-Xiccato et al. 2020a).

Similar to what has been observed for behavioral traits, 2 recent works have suggested that physiological and life-history traits might also be linked to cognitive traits. Cortese et al. (2024) found that European minnows (*Phoxinus phoxinus*) with higher metabolic rates performed better in a spatial task. De Russi et al. (2024a) found that individual European eels (*Anguilla anguilla*) with higher increase in metabolism under stress were slower to learn a problem-solving task. Therefore, physiological and life-history traits might also be involved in the maintenance of cognitive variance, as previously discussed for behavioral traits. It is important to consider a more comprehensive picture of individual differences, including traits from behavioral, physiological, and life-history domains, to best capture all facets of the covariations affecting cognitive variance (De Russi et al. 2024a, 2025).

In this study, we investigated the covariation of cognitive traits with behavioral, physiological, and life history traits in a teleost fish species. We focused on 5 cognitive traits: motor and visual lateralization, which is the asymmetric processing of information between the 2 brain hemispheres (Sovrano et al. 2001; Izvekov and Nepomnyashchikh 2010); spatial learning in a T maze (Lucon-Xiccato et al. 2022); cognitive flexibility measured with a spatial reversal learning task (Miletto Petrazzini et al. 2017; De Russi et al. 2024b); and spatial memory (Tierney and Lee 2011). We additionally considered behavioral traits related to activity, exploration, boldness, and sociability; metabolic rate under basal and stress conditions as physiological traits; and growth rate as a life-history trait. As the study species, we used the tench *Tinca tinca*, assaying wild-caught individuals, as opposed to most studies in this line of research that exploited laboratory fish (Lieggi et al. 2019; Piferrer and Ribas 2020; Näslund 2021; Fox et al. 2024). The use of wild-caught individuals is expected to provide more informative results as these animals likely show individual differences due to both genetic (Armbruster and Schwaegerle 1996; Mackay et al. 2009) and environmental causes (Forsman 2015; Reid and Acker 2022). Following De Russi et al. (2025), we used network analysis to qualitatively and quantitatively explore the relationships among multiple traits from different domains (Gao et al. 2015; Borsboom et al. 2021; Bastelica et al. 2023), thereby offering an improvement over earlier studies that typically focused on only a few traits at a time.

## Materials and methods

### Subjects

**Figure 1:**
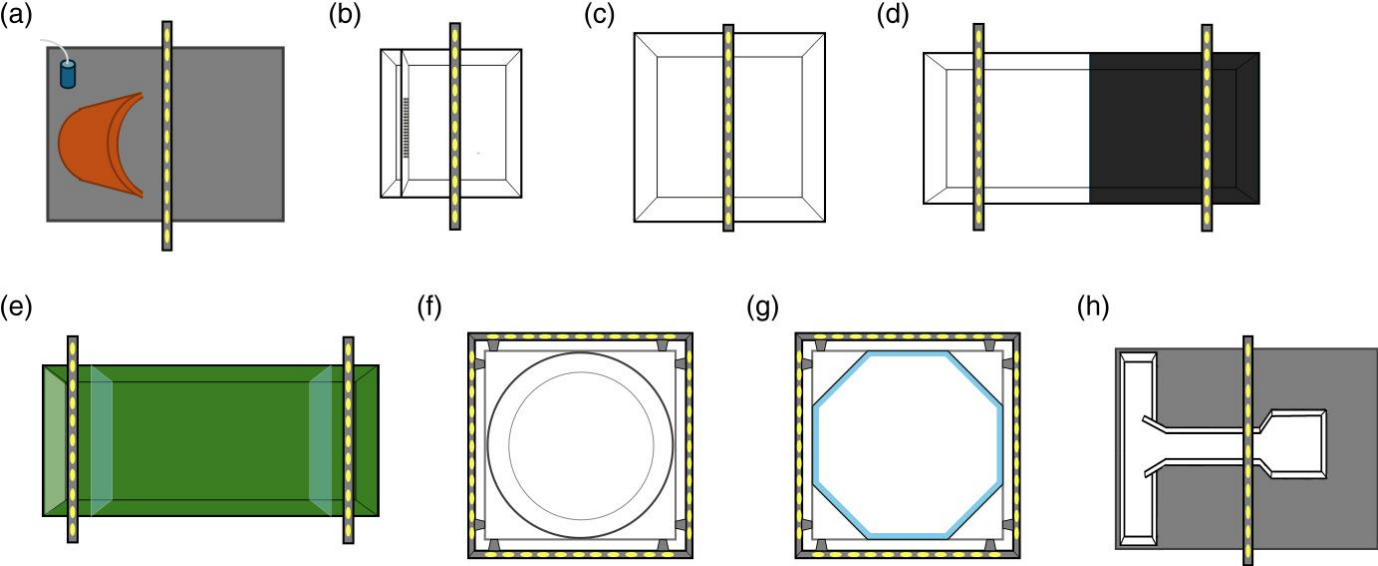
Hounsing tanks and experimental apparatuses. Top view of the housing tanks a) and experimental apparatuses used to assess b) physiology, c) activity and thigmotaxis, d) scototaxis, e) sociability, f) motor lateralization, g) visual lateralization, and h) spatial learning, cognitive flexibility, and memory.

Twenty-four juvenile wild tench were collected with seine net in a private marsh in the Mantua province, Italy. The sample size was determined by the fact that the tench is endangered this part of its native range (Castaldelli et al. 2013; Bani et al. 2021) and based on previous studies with our approach (De Russi et al. 2024a, 2025), was expected to be sufficient to highlight significant network structures. During the first part of the study, the tench were housed in pairs in gray plastic tanks (40 × 30 × 22 cm; water level: 15 cm; Fig. 1a). This setup was chosen because pilot observations indicated that housing in pairs reduced stress levels while simultaneously allowing us to recognize each subject due to slight variations in size and individual markings. Each tank was equipped with an aerator and a shelter (a terracotta pot cut in half: 16 × 14 × 8 cm). The water was maintained at 15 ± 1 °C. Illumination during the day was provided by white LED strips (TMR, distributed by ELCART, Italy; 1,180 lux, 9.322 W/m^2^). For the spatial learning task, the pairs were moved to larger tanks (60 × 40 × 32 cm; water level: 25 cm) that also contained the testing apparatus. These tanks were equipped with an aeration system, shelter (half terracotta pot), and natural gravel at the bottom. The subjects were fed bloodworms (Amtra, Castronno, Italy) once per day. The tanks were cleaned, and the water was partially changed (30%) every 3 days.

Experiments were conducted in accordance with the ABS/ASAB “Guidelines for the treatment of animals in behavioral research and teaching”, the ethical guidelines of the International Society for Applied Ethology, European Legislation for the Protection of Animals used for Scientific Purposes (Directive 2010/63/EU), and the law of the country in which they were performed (Italy, D.L. 26/2014). The experimental protocols have been revised and approved by University of Ferrara's Ethical Committee (permit TLX-2023_1). The health of the subjects was monitored daily by experienced experimenters authorized by the relevant Ministry, and by a certified veterinarian when needed. During the study, 3 subjects showed initial signs of disease (ie, clear patches on the skin). Their testing was immediately interrupted, and the fish were treated to support recovery. At the end of the study, all subjects were released at the sampling site.

### Life-history measurements

Subjects’ length and weight were measured upon collection and at the end of the study. These 2 measurements were used to compute an index of subject's growth rate: ([Final score − Initial score]/Initial score) × 100.

### Physiological traits

Based on prior studies (Queiroz and Magurran 2005; Millidine et al. 2008; Frisk et al. 2012), fish metabolic rate was estimated indirectly using ventilation rate as a proxy. This can be measured as the frequency of operculum movements, which reliably reflects metabolic activity. The subjects were collected from their housing tank with a net and placed in jar filled with water. They were then moved to the experimental room and released into an apparatus for the test (20 × 20 cm; water level: 8 cm). The apparatus was built to favor the observations necessary: it was white and empty, and it was illuminated from above by a white LED strip (TMR, distributed by ELCART, Italy). It included a small lateral compartment (Fig. 1b) with an aerator to ensure stable oxygen levels in the water. This lateral compartment was not accessible to the subject but water exchange with the main compartment was possible thanks to a grid partition. A video camera (HDR-CX405, Sony Corporation, Japan) recorded the subjects from above to observe operculum movements, allowing us to measure ventilation rate.

We measured ventilation twice. The first recording began upon transferring the subject to the novel apparatus. Given the short exposure to the new environment before this recording, the first measure was considered indicative of ventilation, and thus metabolism, under stress conditions. The second recording was conducted the following day, after leaving the subjects in the apparatus overnight. This was considered a measure of resting ventilation rate and thus basal metabolism. Following previous studies (Frisk et al. 2012; Mikheev et al. 2014; Nedelec et al. 2016; De Russi et al. 2025), we recorded ventilation rates for 2 min, an interval that allowed us to capture a specific time point (eg, upon entering the new environment).

We recorded the number of operculum beats per minute (bpm) by playing back the recordings on a computer with the VLC media player software (https://www.videolan.org/vlc/index.it.html). The ventilation rate from the second recording (basal condition) was normalized for the animal's weight before analyses. The ventilation rate from the first recording was used to calculate a relative index of ventilation increase from the basal condition due to stress (hereafter, stress metabolism): ([initial ventilation rate − final ventilation rate]/final ventilation rate) × 100.

### Behavioral traits

We administered an open field test, a scototaxis test, and a sociability test. These tests were chosen primarily because previous research has highlighted their reliability across a number of fish species. Due to the difficulty of obtaining tench specimens prior to the main experiment, since this species faces a serious risk of local extinction, we were unable to perform pilot studies and had to rely on the information on other species already present in the literature. The behavioral tests were performed sequentially in the same day and repeated twice with a 1-week interval. This allowed us to assess whether the trait was repeatable, as in case of personality traits (Bell et al. 2009; Biro and Stamps 2015), and to use the average score as a more reliable estimate of individual performance in the case of traits with low stability.

#### Open field test

Following previous studies (Lucon-Xiccato et al. 2020b; Horka et al. 2024), each subject was individually released in the center of a 40 × 40 × 15 cm unfamiliar arena made of white plastic (water level 8 cm; Fig. 1c). The arena was illuminated from above by white LED strips (TMR, distributed by ELCART, Italy). The subjects were left to explore the arena for the following 30 min and their behavior was recorded by a video camera on the ceiling (HDR-CX405, Sony Corporation, Japan). The Ethovision software (Noldus Information Technology, Wageningen, the Netherlands) was then used to track the subjects’ position from the video recordings and to calculate 2 behavioral variables. The first variable was locomotor activity measured as the distance moved (cm). The second variable, thigmotaxis, was measured as the time spent in the outer sector of the arena, which, based on earlier studies, we defined as 4.5 cm from the edge, roughly corresponding to 1 body length (Schnörr et al. 2012; Johnson and Hamilton 2017; Kyriakou et al. 2017; Caioni et al. 2024). We operatively defined these variables as indicators of exploration tendency due to the fact that the testing was conducted in a novel environment. More explorative individuals were expected to be more active (DePasquale et al. 2022) and spend less time along the edge of the arena (Dos Santos et al. 2023). However, it should be noted that in other disciplines these variables are often interpreted slightly differently. For instance, thigmotaxis can be considered an indication of shyness and anxiety-like behavior (Hamilton et al. 2021). We calculated thigmotaxis scores as: [time spent in the outer sector/total time] × 100. If subjects behaved randomly, we would expect their thigmotaxis score not to deviate from chance (population mean = 40%, corresponding to the outer area divided by the total area of the arena).

#### Scototaxis test

Following Maximino et al. (2010), the scototaxis test was conducted in an apparatus (56 × 15 × 32 cm; water level: 8 cm) half white and half black (Fig. 1d). Two white LED strips (TMR, distributed by ELCART, Italy), placed above each sector, provided the illumination for this test. To begin the test, the subjects were placed individually in the center of the apparatus, and then left undisturbed for 30 min. A camera (HDR-CX405; Sony Corporation, Japan) recorded the subjects’ behavior starting immediately after the transportation. The recordings were played back on a computer and scored with the “CiclicTimer” software, which consisted of a series of independent chronometers activated by the computer keyboard. The software allowed the experimenter to measure the time spent in the white sector and the time spent in the black sector. The fish were expected to generally avoid the white sector as it should be perceived as more dangerous (scototaxis behavior; Maximino et al. 2010). Bolder subjects were expected to visit more often the white sector (Maximino et al. 2010). We calculated scototaxis scores as: [time spent in the darker area/total time] × 100. If subjects behaved randomly, we would expect their scototaxis score not to deviate from chance (population mean = 50%, corresponding to the dark area divided by the total area of the arena).

#### Sociability test

For the sociability test, we followed the paradigm used in Cattelan et al. (2017). Each subject was individually moved in a 60 × 20 × 36 cm apparatus (water level 8 cm) divided into 3 sectors by transparent dividers (Fig. 1e). The subjects were placed in the central sector. One of the 2 lateral sectors was empty. The other lateral sector contained a mirror to simulate the presence of a conspecific (ie, the mirror image of the subject). More social individuals were expected to spend more time near the lateral sector containing the mirror. As for the other tests, illumination was provided by 2 white LED strips placed above the lateral sectors (TMR, distributed by ELCART, Italy) and subject's behavior was recorded from above with a camera (HDR-CX405; Sony Corporation, Japan). The Ethovision software was then used to extract from the recording the time spent in proximity (ie, within 4.5 from the transparent dividers) to the social stimulus and to the empty sector. We computed sociability as: (time spent near the mirror/[total time − time in the central sector]) × 100. If subjects behaved randomly, we would expect their sociability score not to deviate from chance (population mean = 50%, corresponding to the area near the stimulus divided by the sum of the area near the 2 lateral sectors).

### Cognitive measurements

We performed 5 cognitive tests, selected using the same literature-based approach applied for the behavioral tests. The 5 cognitive tests were conducted sequentially. The 2 lateralization tests (motor and visual) were conducted respectively after the behavioral tests and the completion of the cognitive flexibility test and then repeated after 1 week. The remaining 3 cognitive tests had different lengths according to the performance of the subjects (see details below).

#### Lateralization tests

We carried out 2 lateralization tests. The first test focused on the spontaneous rotational preference of the subject in a circular tank (Bisazza and Vallortigara 1997; Izvekov et al. 2014). It was conducted in a circular apparatus (Ø = 40 cm; water level: 8 cm; Fig. 1f) made of white plastic. In this apparatus, the fish typically tend to swim either in a clockwise or counterclockwise direction. Given the absence of evident visual stimuli, we considered this test to be a measure of motor lateralization. The second test focused on lateralization in observing a visual social stimulus (ie, the subject's mirror image; De Santi et al. 2001; Messina et al. 2024). The mirror test was conducted in an octagonal apparatus with mirror walls (mirrors’ size: 17 × 17; water level: 8 cm; Fig. 1g). In this setting, the fish spend most of their time near a mirror, actively swimming against it in an attempt to reach their mirror image. We used a mirror as the social stimulus to reduce the number of wild fish collected for the experiments and to avoid the potential influence of a live conspecific's behavior on that of the focal fish. As fish tend to observe the stimulus using a specific eye at a time, their swimming behavior often develops into alternating clockwise and counterclockwise movements. By quantifying the amount of time spent swimming in each direction, it is possible to assess any preference for using a particular eye to view the stimulus. This, in turn, allows for the determination of lateralization in visual processing.

Illumination for the lateralization apparatuses was provided by white LEDs (TMR, distributed by ELCART, Italy). For both lateralization tests, the subjects were individually released in the center of the apparatus, and their behavior was recorded for 30 min with a camera (HDR-CX405; Sony Corporation, Japan). Each test was repeated twice. Lateralization scores were not expected to be highly repeatable, as this trait is not typically classified as a personality trait and has been shown to exhibit low repeatability (eg, Penry-Williams et al. 2022) and highly plasticity (reviewed in Bisazza and Lucon-Xiccato 2025). By repeating the test and averaging the 2 measurements, we aimed to reduce the influence of short-term fluctuations or situational noise (eg, momentary stress or shifts in attention), under the assumption that such factors would cause individual estimates to vary around a central underlying value. Therefore, averaging the 2 trials was expected to improve the reliability of the estimate, although we acknowledge that additional repetitions would further increase reliability. To address this point, we also conducted a control analysis using only the first trial.

The recordings of the 2 lateralization tests were then analyzed using the “Ciclic Timer” software calculating the following variables: the time spent in the center of the arena (at least at 4.5 cm from the wall in the rotational test and from the mirror in the mirror test); the time spent in the edge swimming in clockwise direction; the time spent in the edge swimming in a counterclockwise direction; and time spent freezing in the edge.

From these measurements, we obtained 2 lateralization indexes for each test (Bisazza and Vallortigara 1997; Bisazza et al. 2000): the relative lateralization index, which considered both the strength and the direction of lateralization and was computed as: ([time spent swimming clockwise − time spent swimming counterclockwise]/[time spent swimming clockwise + time spent swimming counterclockwise]) × 100; and the absolute lateralization index, which only considered the strength of lateralization and was calculated as the absolute value of the relative lateralization index. If subjects didn’t present lateralization, we would expect their lateralization score not to deviate from chance (population mean = 0%, score obtained in subjects spent the same time swimming clockwise and counterclockwise).

### Spatial learning test

The subjects were then tested in a T-maze to assess their spatial learning abilities (Wang et al. 2020; Lucon-Xiccato et al. 2022). Over a series of sequential trials, the subjects had to learn to choose the correct arm of the maze to return to their home tank. The maze was inserted in a larger tank that housed the subjects during this experiment (Fig. 1h). The maze consisted of a start box (12.5 × 14.5 cm), a corridor (18.5 × 7.0 cm), and a choice sector leading to 2 identical arms (15.5 × 7.0 cm; Fig. 1h). The water level inside the maze (3.5 cm) was significantly lower than the rest of the tank because previous studies and various pilot experiments demonstrated that this motivates fish to exit the maze. We chose the specific water level for this study as it was the minimum depth that still allowed the fish to swim without their dorsal fins breaking the surface. Each arm ended with a door, hidden to the subject when it was in the choice sector. The incorrect door was completely closed with a grid net. The correct door was blocked by a net that could be opened by pushing it, and therefore, it allowed the subject to escape the maze and return to the house tank, where it could find shelter and the social companion as the reward, the other door. Both doors were closed outside the trials to prevent accidental exploration of the T-maze by the subject. The correct door (left or right) was randomly assigned to each subject and did not significantly affect spatial learning performance (Wilcoxon rank sum test: *W* = 93.5, *P* = 0.216).

Each subject underwent 6 daily trials, divided into 2 sessions. The intersession interval was 3 h, and the interval between trials in the same session was 30 min. In each trail, the subject was collected with a net and gently placed in the start box of the T-maze. It was then let free to explore the maze it until it exited through the correct door. We recorded the subject's initial choice (correct arm or incorrect arm) as the measure of learning. The trials were considered invalid if not solved in 20 min. These invalid trials were not considered in the analyses and repeated after the aforementioned intertrial interval (30 min). In line with earlier studies (Gatto et al. 2021; De Russi et al. 2025). Montalbano et al. (2022), the task was considered learned when subjects reached the criterion of 10 correct choices out of 12 trials over 2 days (chi-squared test: χ^2^ = 10.667, *P* = 0.001). We used a learning criterion instead of training subjects to a fixed number of trials because this approach helps avoid overtraining, which could affect performance in the subsequent reversal learning task (Mackintosh et al. 1966; Nakagawa 1992).

#### Cognitive flexibility test

After reaching the criterion in the spatial learning test, the rewarded arm was switched to perform a reversal learning test (Miletto Petrazzini et al. 2017). Therefore, the subject had to learn to revert the choice between the 2 arms learned in the previous test. We continued the reversal learning test until the fish reach then same criterion of the learning task. Cognitive flexibility performance was calculated as: ([days to criterion in the reversal learning test − days to criterion in the learning test]/days to criterion in the learning test). This calculation provided an index of individual differences in flexibility, corrected for individual differences in learning (De Russi et al. 2024b). The negative sign at the beginning of the formula ensures that the index assigns higher values to subjects with greater cognitive flexibility.

#### Spatial memory test

Two months after completing the reversal learning test, we performed a memory test based on re-training. The length of the interval was based on studies in other species, which suggest that fish can easily retain learned information for several days (Moreira et al. 2025) and, in some cases, can retrieve it even after many months (Triki and Bshary 2020). We chose an intermediate interval of 2 months to ensure that some memory trace would be detectable, while keeping the task sufficiently challenging to reveal differences among individuals. We tested the subjects in same T-maze, where the rewarded arm was the same as the one in the previous reversal learning test. The testing continued until the subject reached the aforementioned learning criterion. We then calculated a memory index as: ([days to criterion in the memory test − days to criterion in the reversal learning test]/days to criterion in the reversal learning test). The negative sign at the beginning of the formula ensures that the index assigns higher values to subjects with greater memory. We used reversal learning performance as the baseline because reversal learning is known to influence discrimination performance in this species (Lucon-Xiccato and Bisazza 2014) and in others (eg, Mackintosh et al. 1968). Accordingly, while we will refer to this as the memory test for simplicity, it is also possible that, rather than measuring memory per se, it reflects a progressive improvement in understanding the task due to repeated training.

### Statistical analysis

Data analysis was performed using RStudio (ver. 2023.12.1 + 402). Missing data (1.79% of the dataset; 6 missing observations from 3 subjects out of 336 observations) were due to subjects showing signs of disease (see Subjects section). In detail, 1 subject did not complete the visual lateralization, cognitive flexibility and spatial memory tests, while 2 additional subjects did not conclude the spatial memory test.

To compare the different trials for behavioral and lateralization traits and the different timepoints for physiological and life history traits, we used paired-samples *t*-test (“*t.test*” R function). Before running these tests, homogeneity of variances was tested with the Levene's test (“*leveneTest*” R function). For the lateralization indexes, we used 1-sample *t*-test (“*t.test*” R function) to compare the observed score against the score attended in case of no lateralization (ie, lateralization index = 0%). The same test was used to compared scores against random choice (eg, thigmotaxis test: expected population mean = 40%; scototaxis test and sociability test: expected population mean = 50%; cognitive flexibility and memory index: expected population mean = 0). Paired-samples *t-*test was used to compare day to criterion in the different spatial tasks, while a binomial test (“*binom.test”* R function) was used to assess whether the performances recorded during the first day of the memory test were statistically higher than chance indicating that subjects remember the previously learned task. To assess repeatability in behavioral and lateralization tests, we used the “*rptR*” R package (Stoffel et al. 2017).

To interpret subjects’ performance (ie, number of days to criterion) in the spatial learning, reversal learning, and memory tests, we simulated the performance of a set of subjects choosing randomly between the arms of the T-maze (De Russi et al. 2024b). The set of simulated subjects had the same sample size of the real subjects’ sample that participated in the tasks. For each simulated subject, we used a random binomial function to calculate the number of correct responses in 6 daily trials with 50% probability of success. This was done for X sets of trials, where X corresponded to the maximum number of days to reach the criterion observed in our subjects (see Results section). We then run *n* = 10,000 replicates of the simulation and for each replicate, we computed the percentage of simulated subjects that reached the learning criterion. Then, we calculated a *P*-value indicating the probability of obtaining the observed number of successful subjects in the replicates of the simulated experiment. A significant *P*-value would indicate that the observed performance could not be explained by subjects randomly choosing between the 2 arms of the T-maze. Finally, we carried out an analysis that also took into account the performance improvement across testing days. Since individual's responses followed a binomial distribution, we performed a logistic regression by grouping the number of errors and the number of correct choices in each day (minimum value = 0, maximum value = 6 for each variable) in a matrix (“*cbind*” function). We used a Generalized Linear Mixed-effects Model (GLMM; “*glmer*” R function, “*lme4*” package) with binomial error distribution. Testing day and subject ID were fitted as fixed and random effect, respectively.

To analyze the covariation between the various measures obtained from the different tests (which will be referred from now on as “traits”), we first used a pairwise correlation analysis. For behavioral traits and lateralization, we used the average value obtained from the 2 trials as final score, while for spatial learning test we used the days needed to reach criterion. Using the Spearman rank test (“*cor.test*” R function), we compiled a correlation matrix containing the correlation coefficients for all possible pairs of traits. We imputed the missing data using the “*mice*” package (van Buuren and Groothuis-Oudshoorn 2011). We performed repeated multiple imputations (argument *m* = 5) with the number of interactions set to 50 (argument maxit = 50). Univariate imputation method of predictive mean matching (argument meth = “pmm”, seed = 500) was finally used for imputing missing data.

To further investigate associations between traits, we applied a network analysis. This method allowed us to obtain a more comprehensive evaluation of the relationship between traits not limited to pairwise associations as the correlation analysis. In the network, each trait represented a node and associations across them (ie, edges) were corrected for the influence of all the other nodes. This limits the problem of spurious correlation (ie, when 2 variables are connected through a third one and not directly) of the simple correlation approach (Epskamp et al. 2018; Epskamp and Fried 2018). We estimated correlation network structure with the “*estimateNetwork*” function (bootnet package; Epskamp et al. 2018). The correlation analysis used to estimate the network structure was obtained with the “*cor*” function from the “psych” package (argument default = cor; argument corMethod = “cor”; argument corArgs = “spearman”). We obtained both an unweighted network, which consider only the association between nodes with weight significantly different from zero (“igraph package”; Csárdi et al. 2024) and a weighted network including all possible associations. To confirm the stability of the significant relationships that emerged, we estimated edge weight stability through nonparametric bootstrap (resampling rows with replacement; “bootnet” package; samples = 1,000; Epskamp et al. 2018). As confirmation of the main results, we also calculated an additional network using alternative variables: the raw data on stress metabolism (operculum-beating frequency), the raw data on cognitive flexibility (number of days to reach criterion in the reversal learning phase), and the data from only the first lateralization trial.

To describe the importance of nodes in the network's structure, we focused on 3 centrality metrics. The first metric was the eigenvector, which reflects a node's connection within the network based on its strength and the strength of the connections of its neighbor nodes (Whitehead 2008). The second metric was the strength of the node, which is the sum of the absolute value of the edge weights connected to a certain node (Whitehead 2008; Robinaugh et al. 2016). However, these 2 metrics can mask the effect of negative interaction in the network. For this reason, we also considered the expected influence metric, which consider differently positive and negative edges within the network (Robinaugh et al. 2016). Higher values of the expected influence metric are interpreted as stronger importance of the node in the network. To confirm the accuracy of the computed centrality metrics describing the networks’ structure, we followed the suggested approach by Epskamp and colleagues (2018). By calculating the centrality measures of interest considering increasingly smaller samples of the original data, the analysis assessed the correlation between the original centrality measures and those calculated via bootstrap, a framework defined as case-dropping subset bootstrap. Then, we computed the correlation stability coefficient (CS-coefficient, “*corStability*” function), a measure indicating the percentage of the data that can be reasonably dropped to retain with 95% certainty a correlation of 0.7. This threshold (*r* = 0.7) has been chosen as, in behavioral science, it can be considered an indicator of large effect (Cohen 2013). It has been suggested that the CS-coefficient should not be below 0.25. This helped us identifying the metrics that were stable enough to describe the network in spite of the sample size.

## Results

### Life history traits

At the beginning of the study, tench length and weight were 5.82 ± 0.35 cm (mean ± SD) and 1.82 ± 0.39 g, respectively. At the end of the study, length and weight were 6.11 ± 0.37 cm and 2.29 ± 0.50 g. As length and weigh were consistently and strictly correlated (Spearman's rank correlation test: first measurement, *ρ* = 0.804, *P* < 0.001; second measurement, *ρ* = 0.743, *P* < 0.001), we used only the length to calculate the growing rate used in the following analysis. The average length growth index in the population was 5.23 ± 4.01%, suggesting marked differences between individuals (Figure S1a).

### Physiological traits

The ventilation rates decreased significantly from the stress (313.96 ± 82.85 bpm) to the basal condition (171.83 ± 51.09 bpm; paired-samples *t*-test: *t*_23_ = 6.077, *P* < 0.001). The basal ventilation corrected for the subjects’ weight (considered as proxy of basal metabolism) was 84.50 ± 25.14 bpm/g (Figure S1b). The increase in ventilation rates due to stress (stress metabolism) was 102.68 ± 88.54% (Figure S1c).

### Behavioral traits

In the open field test, the average distance covered (activity) by the subjects was 4,053.14 ± 2,160.74 cm (Figure S2a). Subjects preferred the edge of the arena (78.26 ± 18.27%; 1 sample *t*-test: *t*_23_ = 9.455, *P* < 0.001; Figure S2b), showing the expected thigmotactic behavior. There were no significant differences between the 2 trials for both traits (paired-samples *t*-test: activity, *t*_23_ = 0.464, *P* = 0.647; thigmotaxis, *t*_23_ = 0.101, *P* = 0.920). The 2 traits were significantly repeatable across the 2 trials (repeatability estimation via parametric bootstrapping: activity, *R* = 0.645 [0.312; 0.818], *P* = 0.001; thigmotaxis, *R* = 0.778 [0.561; 0.900], *P* = 0.001).

In the scototaxis test, the subjects showed a significant preference for the black sector of the apparatus (90.68 ± 7.77%, 1 sample *t*-test: *t*_23_ = 25.636, *P* < 0.001; Figure S2c). This scototaxis behavior did not vary between trials (paired-samples *t*-test: *t*_23_ = 1.620, *P* = 0.119) and was not significantly repeatable (repeatability estimation via parametric bootstrapping: *R* = 0.220 [0; 0.552], *P* = 0.149).

In the sociability test, the subject did not show an average significant preference for the social stimulus (54.94 ± 21.45%, 1 sample *t*-test: *t*_23_ = 1.128, *P* = 0.271; Figure S2d). Subjects’ social behavior did not vary between trials (paired-samples *t*-test: *t*_23_ = 0.421, *P* = 0.677) and was not significantly repeatable (repeatability estimation via parametric bootstrapping: *R* = 0.099 [0; 0.489], *P* = 0.303).

### Cognitive traits

#### Lateralization

In the rotational test, the average relative lateralization index observed was −2.40 ± 33.93% and was not different from zero (1 sample *t*-test: *t*_23_ = 0.347, *P* = 0.732; Figure S3a), suggesting no population bias in the direction of motor lateralization. There was no difference between trials (paired-samples *t*-test: *t*_23_ = 0.961, *P* = 0.346) and no significant repeatability (repeatability estimation via parametric bootstrapping: *R* = 0.281 [0; 0.609], *P* = 0.091). The absolute lateralization index in the rotational test was significantly different from zero (26.15 ± 21.06%; 1 sample *t*-test: *t*_23_ = 6.085, *P* < 0.001; Figure S3b). The absolute index did not differ between trials (paired-samples *t*-test: *t*_23_ = 1.716, *P* = 0.100) and was not repeatable (repeatability estimation via parametric bootstrapping: *R* = 0.211 [0; 0.552], *P* = 0.150)

The relative lateralization index in the mirror test was −1.18 ± 15.56% and did not differ from zero (1 sample *t*-test: *t*_22_ = 0.364, *P* = 0.719; Figure S3c), suggesting no population bias in the direction of visual lateralization. Considering the relative lateralization index, there was no difference between trials (paired-samples *t*-test: *t*_22_ = 1.953, *P* = 0.064) and no significant repeatability (repeatability estimation via parametric bootstrapping: *R* = 0 [0; 0.410], *P* = 1). The absolute lateralization index in the mirror test was significantly different from zero (12.69 ± 8.68%; 1 sample *t*-test: *t*_22_ = 7.007, *P* < 0.001; Figure S3d). The absolute lateralization index did not differ between trials (paired-samples *t*-test: *t*_22_ = 1.030, *P* = 0.314) and was not significantly repeatable (repeatability estimation via parametric bootstrapping: *R* = 0.205 [0.093; 0.606], *P* = 0.135).

#### Spatial learning

All subjects (24 out of 24; 100%) reached the criterion of the spatial learning task. On average, they required 4.83 ± 2.12 days, with range 2 to 8 days. The simulation showed that this performance could not be achieved by chance (percentage of simulated subjects reaching the criterion within 8 days: 11.39 ± 6.55%; *P* < 0.001). In support of this, a repeated measures model uncovered a significant decrease in the probability to commit errors across the days of training (GLMM: *β* = −0.288, *χ*^2^_1_ = 29.884, *P* < 0.001; Figure S3e), evidencing that fish progressively learned the task.

#### Cognitive flexibility

For the reversal learning task, all subjects involved reached the criterion (23 out of 23; 100%). Overall, they needed 5.13 ± 1.55 days, with range 3 to 8 days. The simulation revealed that this performance could not be obtained by chance (percentage of simulated subjects reaching the criterion within 8 days: 15.59 ± 6.59%; *P* < 0.001). The repeated measures analyses uncovered a significant decrease in the probability to commit errors across days (GLMM: *β* = −0.397, *χ*^2^_1_ = 61.376, *P* < 0.001; Figure S3f). The flexibility index for the population was −0.41 ± 0.99 (index range: −3.00, +0.63) and it was not significantly different from zero (1-sample *t*-test: *t*_22_ = 1.979, *P* = 0.061). This indicated that subjects took a similar number of errors to reach the criterion in the learning and the reversal learning test.

#### Spatial memory

All subjects (21 out of 21; 100%) reached the criterion in the memory task. On average, they required 2.86 ± 1.01 days, with range 2 to 5 days, with performances significantly already higher than the chance during the first day of testing (Exact Binomial Test: *P* < 0.001, CI 0.65, 0.81). The simulation showed that this performance could not be achieved by chance (percentage of simulated subjects reaching the criterion within 5 days: 16.29 ± 5.49%; *P* < 0.001). The repeated measures model uncovered a significant decrease in the probability to commit errors across the days of testing (GLMM: *β* = −0.653, *χ*^2^_1_ = 17.557, *P* < 0.001; Figure S3g), evidencing that fish progressively learned the task. The subjects took less time to complete the memory test compared with the reversal learning task (paired-samples *t*-test: *t*_20_ = 6.136, *P* < 0.001), indicating that they remembered the learned contingency. The memory index was: 0.39 ± 0.34 (index range: −0.33, +0.71) and was significantly different from zero (1 sample *t*-test: *t*_20_ = 5.206, *P* < 0.001). This indicated that the subjects required fewer days to reach the criterion in the memory test compared with the initial learning phase (paired-samples *t-*test: *t*_20_ = 3.419, *P* = 0.003), thereby suggesting that they either exhibited memory or that performance in previous phases (spatial learning and cognitive flexibility) contributed to an improved ability to learn the task.

### Correlation across traits

**Figure 2:**
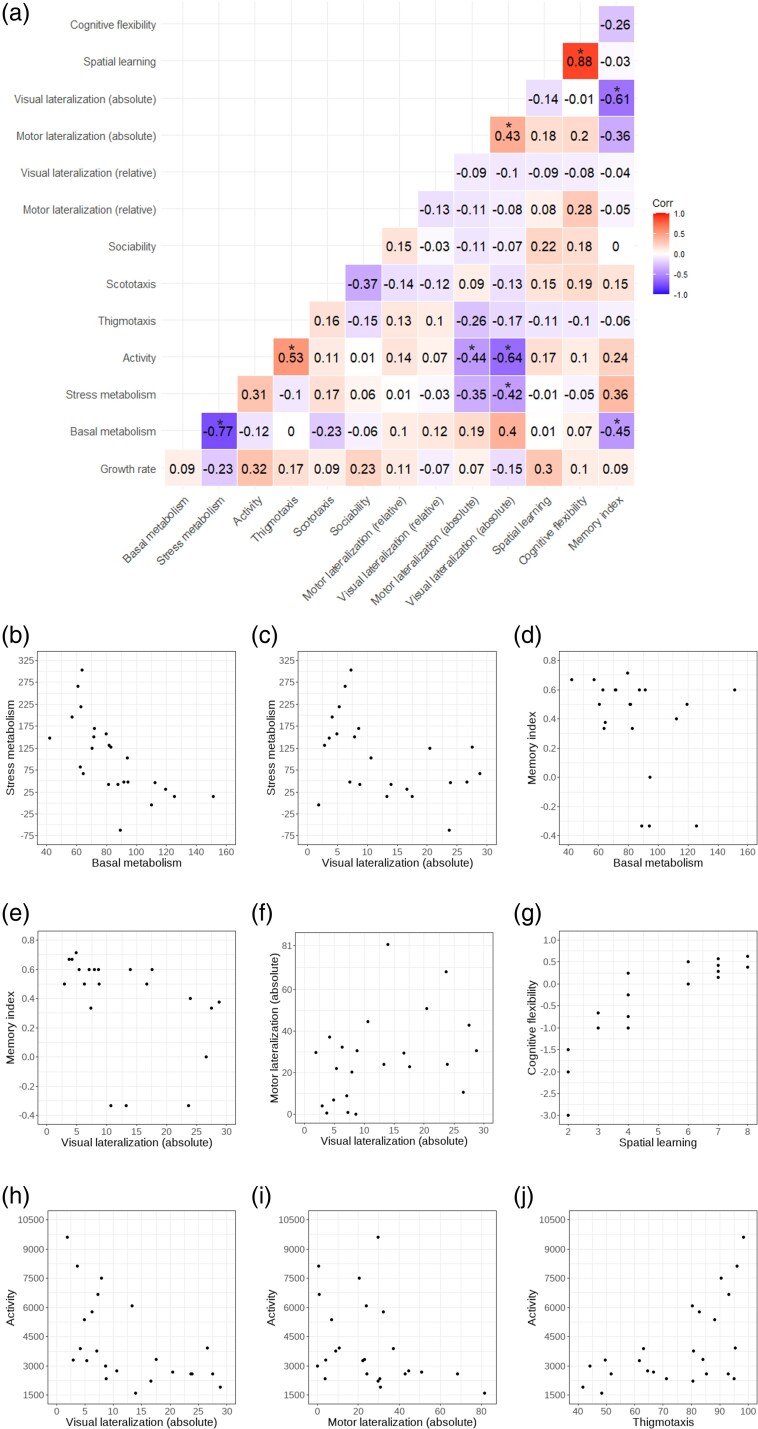
Results of the correlation analysis. a) Correlations matrix reporting Spearman's *ρ*; significant correlations are indicated by an asterisk. b–j) Scatterplots of significant correlations between traits.

The initial correlation analysis showed a complex pattern of covariation between pairs of traits (Fig. 2a). The tench sample displayed 9 significant correlations (Fig. 2b–j). Five of these significant correlations involved traits from the same domain: Basal metabolism—Stress metabolism; Activity—Thigmotaxis; Motor lateralization (absolute)—Visual lateralization (absolute); Visual lateralization (absolute)—Memory index; and Spatial learning—Cognitive flexibility. Four significant correlations involved traits from different domains: Basal metabolism—Memory index; Stress metabolism—Visual lateralization (absolute); Activity—Motor lateralization (absolute); and Activity—Visual lateralization (absolute). Cognitive traits were involved in most significant correlations (7 out of 9). For instance, memory was negatively correlated with basal metabolism, indicating that subjects with lower basal metabolism were better at remembering the learned contingency, and with absolute visual lateralization, suggesting how subject with a stronger cerebral lateralization displayed lower performance in recalling a learned task. Both absolute lateralization indexes (motor and visual) were negatively correlated with activity in the open field test. Note that using raw data the correlation between measures of learning and reversal learning was significant (Spearman's rank correlation test: *ρ* = −0446, *P* = 0.033) and the 1 between stress and basal metabolism neared significance (*ρ* = 0.387, *P* = 0.062).

### Network analysis

**Figure 3:**
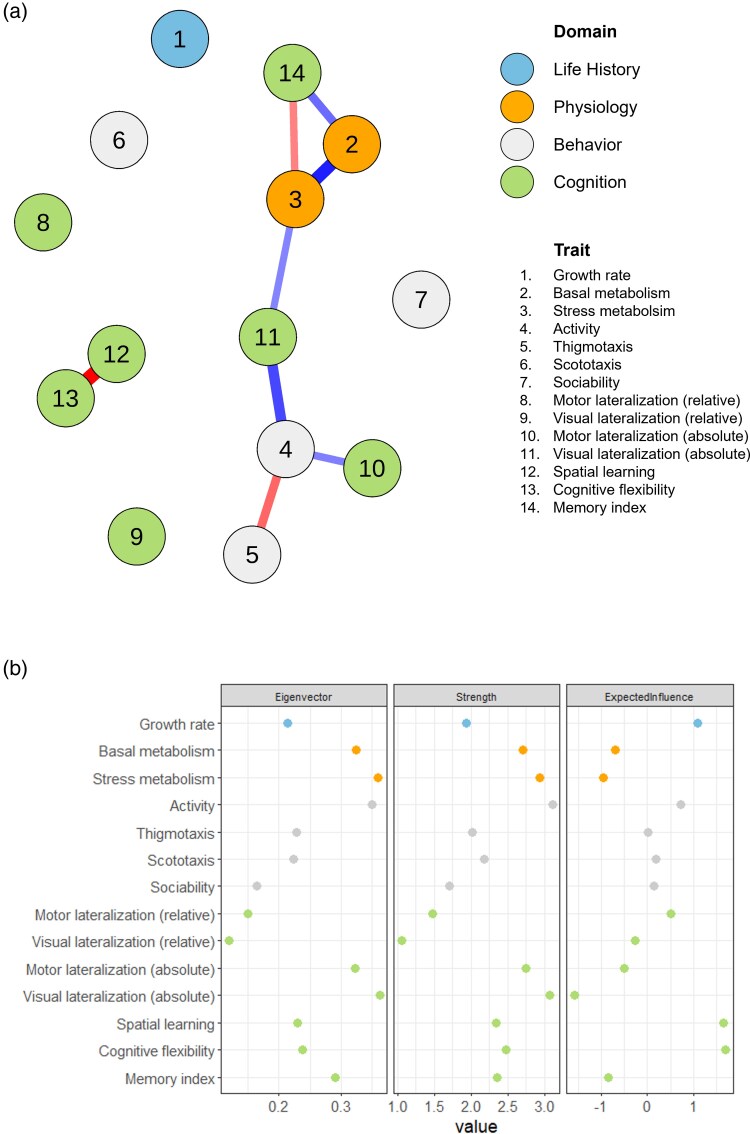
Results of the network analysis. a) Network plot reporting significant associations among traits; each node represents a trait; the color of the nodes represents the domain of the trait (life history = blue; physiology = orange; behavior = light gray; cognition = green); the thickness of an edge (ie, the line connecting 2 nodes) represents the strength of the association between nodes; the color of edge represents whether the direction of the association (negative = blue; positive = red). b) Centrality s5metrics (strength, eigenvector and expected influence) of all network nodes; the 4 domains are differentiated by color (life history = blue; physiology = orange; behavior = light gray; cognition = green).

The unweighted network suggested a central role of cognitive traits connecting different subnetworks and traits from other domains (Fig. 3a). Additional network calculated with the raw data of stress metabolism and cognitive flexibility and the first lateralization trial confirmed the main role of cognitive traits (Figure S4). We identified 9 significant associations among traits, substantially confirming the correlation analysis. Two associations involving cognitive traits previously detected through the correlation analysis were not significant in the network: Visual lateralization (absolute)—Memory index; and Motor lateralization (absolute)—Visual lateralization (absolute). An additional association became significant in the network as compared to the correlation analysis (Stress metabolism—Memory index; regularized partial correlation Table S1). The permutation analysis on the weighted network (eg, the network that included all possible associations) supported the stability of the 8 significant associations (Figure S5). Therefore, these significant associations were key determinants of the network structure.

In the weighted network, we considered 3 centrality metrics that identified the critical nodes in the network's structure (Fig. 3b). Stress and basal metabolism, activity and absolute visual and motor lateralization, and memory showed higher values for the eigenvector measure, indicating that these traits were high-influential and interacted with other highly influential and well-connected nodes. With the exception of memory, these traits also displayed high nodes’ strength, indicating that they presented the stronger covariance levels with the other traits in the network. When looking at the expected influence metric, which considered also the direction of the association (ie, positive and negative), 3 cognitive traits emerged as the most important in the network's structure: spatial learning, cognitive flexibility, and absolute visual lateralization. The permutation analysis used to evaluate the 3 types of metrics suggested that the expected influence was reliable in our dataset (CS = 0.583; Figure S6), while caution should be exerted when considering the strength (CS = 0.125) and the eigenvector metrics (CS = 0.125). These results thus confirm that cognitive traits with high expected influence values are the most important contributors to the covariance structure among nodes in the network and this effect was not likely to be affected the sample size.

## Discussion

We investigated the presence and structure of covariation between cognitive, life-history, physiological, and behavioral traits. We found that some individual differences in cognitive traits were significantly interrelated in our study species, the tench. Moreover, cognitive traits not only covaried with traits from other domains, but had a critical influence on the entire covariation network. These findings suggest that covariation with traits from other domains can determine indirect selection and tradeoffs affecting cognitive traits, contributing to the maintenance of cognitive variance.

Both considering the pairwise correlations and the network analysis, our study substantially confirms the presence of a trait interconnection both within and between domains, which had been hypothesized by earlier works (eg, Carere and Locurto 2011; Bailey et al. 2022). Regarding associations within the cognitive domain, the most notable suggests that individuals’ spatial learning performance was negatively correlated with cognitive flexibility, which is in line with several other studies (Bebus et al. 2016; Tello-Ramos et al. 2019; Morand-Ferron et al. 2022). A recent mesocosm experiment suggested that this negative relationship may be driven by phenotypic plasticity shaped by environmental predictability. Guppies reared in stable environments exhibited enhanced learning abilities but showed limited flexibility. In contrast, subjects exposed to a variable environment during development demonstrated greater cognitive flexibility, likely as an adaptive response to cope with changing conditions, but reduced learning abilities (Lucon-Xiccato et al. 2023). Therefore, differences in the resource predictability experienced by tench in their natural environment prior to the experiments may explain the observed relationship between learning and flexibility.

In the network, which provided a comprehensive view of the relationships between all the traits considered, we found a significant importance of the lateralization. Subjects with strong lateralization displayed a lower metabolic response to stress and were less active in the exploration test. This result is supported by studies in humans and captive animals, in which lateralization and response to stress appear interconnected (Ocklenburg et al. 2016; Goma et al. 2023; Rogers 2023), as well as by several studies reporting correlations between lateralization and behavior (eg Irving and Brown 2013; Goursot et al. 2019). When correlations involving visual lateralization were examined across the entire network (eg, Figure 3), this trait emerged as a crucial bridge between subnetworks involving life-history, metabolism, and behavior. Alterations due to selection or plasticity in individual differences in visual lateralization (Brown et al. 2007; Broder and Angeloni 2014; Lucon-Xiccato et al. 2016; Panizzon et al. 2024) are therefore expected to cause significant alterations in alterations in the overall connectivity between behavioral and physiological domains. Motor lateralization was still included in the network, albeit with a less prominent role. Notably, although our measure of visual lateralization may have been influenced by motor lateralization due to the similarity of the tests, we did not find a direct link between the 2 traits, suggesting that our measures primarily captured distinct aspects of lateralization.

The importance of cognitive traits in the network also emerged from the result of the spatial tasks. Spatial learning and cognitive flexibility were, along with visual lateralization, the traits with the strongest influence in the whole network. Interestingly, memory, which still exerted substantial influence in the network, was not related to spatial learning and cognitive flexibility performance, suggesting that we measured a nonspecific memory function rather than a form of spatial memory. Alternatively, as our memory test was based on a re-training procedure, it is possible that the involvement of other functions could explain this lack of correlation across all the spatial tests. Moreover, this finding further suggests that while cognitive tasks are often influenced by noncognitive confounds (van Horik and Madden 2016), in our case a substantial proportion of the observed performance variance was likely attributable to distinct cognitive functions. If noncognitive factors had dominated, we would have expected strong positive associations among all 3 spatial test performances, which was not the case despite using the same test structure. Importantly, memory performance was significantly linked to both basal and stress metabolism, albeit in opposite directions. Tench with lower basal metabolism, but larger increases in metabolism under stress, displayed greater memory abilities. This could be explained by the marked effects of stress hormones on memory encoding, consolidation and retrieval (Sandi and Pinelo-Nava 2007), often enhancing them in the case of acute stress (Osborne et al. 2015; Sherman et al. 2023) and impairing them under chronic stress (Rezayof et al. 2022).

Overall, this study provides evidence that individual differences across most major domains of animal biology (Williams 2008; Kanai and Rees 2011; Holtmann et al. 2017; Lucon-Xiccato and Bisazza 2017; Laskowski et al. 2022) are not independent, emphasizing the need for an integrative approach to fully understand how animal traits evolve and adapt to the environment. Notably, cognitive traits covaried with life-history, behavioral and physiological traits, playing a significant role in the structure of the covariance network. We speculate that the large influence of cognitive traits may be linked to the high metabolic demands of their underlying neural tissues, which are among the most energetically costly in the organism (Aiello and Wheeler 1995). Indeed, while cognitive abilities can only be assessed indirectly through behavioral observations, such as those used in our study, evidence suggests that they are at least partially related to the development of neural substrates (MacLean et al. 2014; Herculano-Houzel 2017), including in our study species (Kotrschal et al. 2013, 2015; Buechel et al. 2018). Supporting this idea, the relationship we observed between memory and basal metabolism was negative, suggesting the presence of an energetic tradeoff. When such tradeoffs are particularly pronounced, they may influence not only cognitive performance but also have cascading effects on other functional domains, potentially impacting the organism as a whole (Christie and Schrater 2015; Cortese et al. 2024; Tait et al. 2024), as initially proposed for the evolution of primates’ brain (expensive tissues hypothesis: Aiello and Wheeler 1995).

The key role of cognitive traits in the covariance network offers a framework for understanding how individual differences in cognition evolve and are maintained. Selective pressures (Dingemanse and Réale 2005; Penke et al. 2007) and energetic tradeoffs driven by specific environmental demands (Price et al. 2003; Des Roches et al. 2018; Cockrem 2022), which sustain life-history, behavioral and physiological individual differences, can indirectly maintain cognitive variation. Moreover, given the weight of cognition in the entire network, evolutionary changes in cognitive variance may be constrained by other traits, and when they do occur, they likely impact the entire covariation network. Insights into how selective pressures acts on multiple, covarying traits, even from different domains, are beginning to emerge. For instance, a recent study on juvenile European eels found that combinations of traits involving cognition, behavior, and life-history, rather than individual traits alone, predict migration success (De Russi et al. 2024a). More research is needed on this direction. In particular, future analyses should incorporate additional traits to capture aspects not addressed in the present study, such as life-history measures beyond growth (eg, reproductive success, longevity), as well as a broader range of behavioral and cognitive traits. Moreover, future studies should consider exploring species and population-level differences in trait covariation, as well as variations within populations, such as those related to sex (Stamps and Groothuis 2010; Roy and Bhat 2018; De Russi et at. 2024b).

It is important to note that the test for one of the traits, the sociability test, did not yield the expected outcome (eg, a significant preference for the social stimulus). Although we did not observe biting or other overt aggressive behaviors, it is possible that the subjects perceived the mirror stimulus as a rival rather than a social companion (Pham et al. 2012). This suggests that the test may not be suitable or valid for assessing sociability in this species, a possibility we could not verify due to the difficulty of conducting preliminary work with this locally endangered species. Moreover, it should be noted that the main network included a combination of both repeatable traits and nonrepeatable traits such as lateralization. Repeatability analyses are typically used to determine whether a behavioral trait qualifies as a personality trait (Bell et al. 2009; Koski 2011; Brust et al. 2015), but in our case it is not entirely clear how essential this classification is beyond the formal label. For the purposes of our study, what matters more is the robustness of each trait estimate. Several of our measurements were based on 2 trials’ averages, which likely reduced short-term noise, although additional repetitions would certainly improve precision. However, there is also no full consensus on how much averaging 2 measurements improves estimates of variable traits, making it difficult to determine exactly how robust our approach was. To address this, we performed a control analysis using only a single estimate of the nonrepeatable trait (lateralization). The results suggest that the main conclusion of the study, the central role of cognitive traits within the network, remains stable. Note that this analysis also produced a network that was not identical to the one based on the mean value. This highlights an important insight: while repeatability per se may not be the central issue, the choice and treatment of traits can influence specific aspects of the network structure. Because network models are inherently dependent on the collective set of traits included, adding or removing a trait, or using different versions of that trait, can propagate through the system and subtly alter network connections. At present, it remains unclear how strongly these changes affect the overall resemblance of networks or the general conclusions drawn from them. This deserves further investigation as a broad methodological question relevant to the field. Understanding the sensitivity of behavioral networks to trait selection will help clarify the tradeoffs and enhance the interpretative power of this promising approach.

## Supplementary Material

arag027_Supplementary_Data

## Data Availability

Analyses reported in this article can be reproduced using the data and code provided by De Russi et al. (2026).
