# Supplementary material for: Cognitive individual differences are key in the network of trait covariance
Source: Behav Ecol. 2026 Mar 2;37(3):arag027. doi: 10.1093/beheco/arag027 (PMC13017037; doi:10.1093/beheco/arag027)

Cognitive individual differences are key in the network of trait covariance

Table S1

Figures S1-S6

Table S1

Trait	1	2	3	4	5	6	7	8	9	10	11	12	13	14
1	0.000	0.094	-0.230	0.322	0.174	0.092	0.226	0.112	0.012	0.068	-0.191	0.296	0.112	-0.001
2	0.094	0.000	-0.774	-0.118	0.001	-0.234	-0.064	0.096	0.163	0.187	0.357	0.014	0.092	-0.508
3	-0.230	-0.774	0.000	0.315	-0.104	0.172	0.061	0.005	-0.006	-0.351	-0.422	-0.010	-0.045	0.434
4	0.322	-0.118	0.315	0.000	0.526	0.106	0.005	0.137	0.137	-0.443	-0.635	0.173	0.135	0.054
5	0.174	0.001	-0.104	0.526	0.000	0.156	-0.150	0.135	0.025	-0.261	-0.127	-0.114	-0.124	-0.118
6	0.092	-0.234	0.172	0.106	0.156	0.000	-0.375	-0.141	-0.124	0.086	-0.123	0.153	0.174	0.248
7	0.226	-0.064	0.061	0.005	-0.150	-0.375	0.000	0.146	0.051	-0.105	-0.083	0.217	0.203	0.013
8	0.112	0.096	0.005	0.137	0.135	-0.141	0.146	0.000	-0.119	-0.112	-0.086	0.085	0.275	-0.035
9	0.012	0.163	-0.006	0.137	0.025	-0.124	0.051	-0.119	0.000	-0.027	-0.145	-0.078	-0.062	-0.106
10	0.068	0.187	-0.351	-0.443	-0.261	0.086	-0.105	-0.112	-0.027	0.000	0.383	0.179	0.218	-0.324
11	-0.191	0.357	-0.422	-0.635	-0.127	-0.123	-0.083	-0.086	-0.145	0.383	0.000	-0.133	-0.014	-0.366
12	0.296	0.014	-0.010	0.173	-0.114	0.153	0.217	0.085	-0.078	0.179	-0.133	0.000	0.878	-0.005
13	0.112	0.092	-0.045	0.135	-0.124	0.174	0.203	0.275	-0.062	0.218	-0.014	0.878	0.000	-0.147
14	-0.001	-0.508	0.434	0.054	-0.118	0.248	0.013	-0.035	-0.106	-0.324	-0.366	-0.005	-0.147	0.000

Regularized partial correlation between traits. 1-Growth rate; 2-Basal metabolism; 3-Stress metabolism; 4-Activity; 5-Thigmotaxis; 6-Scototaxis; 7-Sociability; 8-Motor lateralization (relative); 9-Visual lateralization (relative); 10-Motor lateralization (absolute); 11-Visual lateralization (absolute); 12-Spatial learning; 13-Cognitive flexibility; 14-Memory index

Figure S1. Variation in life-history and physiological traits. Histograms of (a) growth rate, (b) basal metabolism, and (c) stress metabolism. Bars represent observed data frequency and curves represent probability density functions.

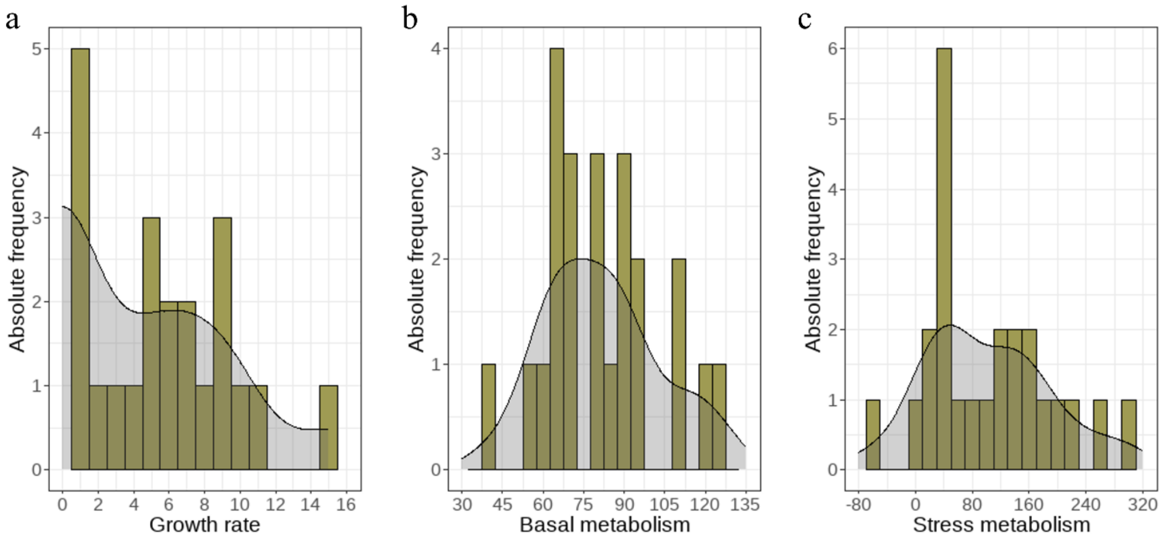

Figure S2. Variation in behavioral traits. Histograms of (a) activity, (b) thigmotaxis, (c) scototaxis, and (d) sociability scores. Bars represent observed data frequency and curves represent probability density functions.

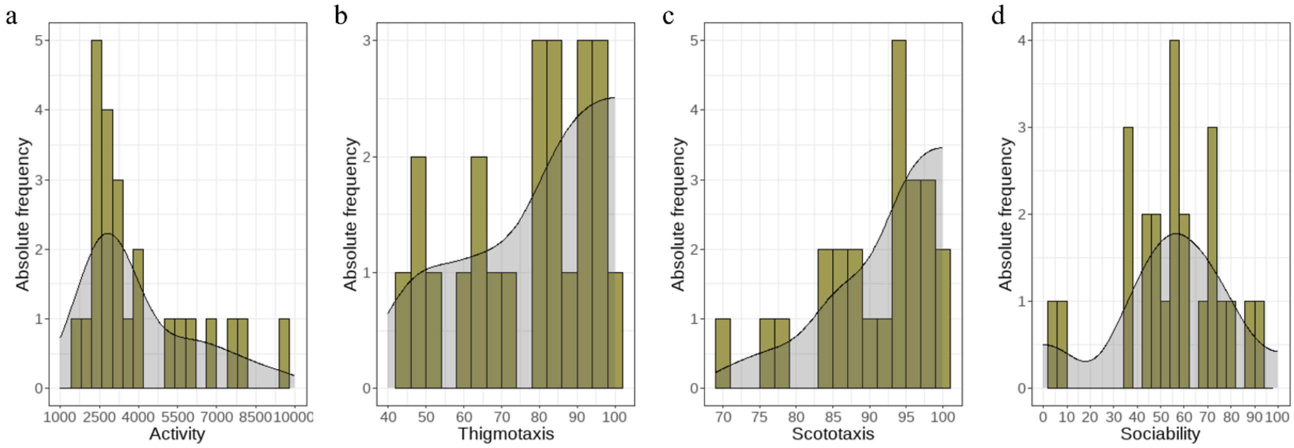

Figure S3. Variation and performance in the cognitive tests. Histograms of relative and absolute lateralization index in the (a, b) motor and (c, d) visual lateralization tests; bars represent observed data frequency and curves represent probability density functions. Number of errors in each day of training in the (e) spatial learning, (f) cognitive flexibility, and (g) memory tests; points and shaded areas represent respectively means and 95% confidence intervals predicted by the GLMMs.

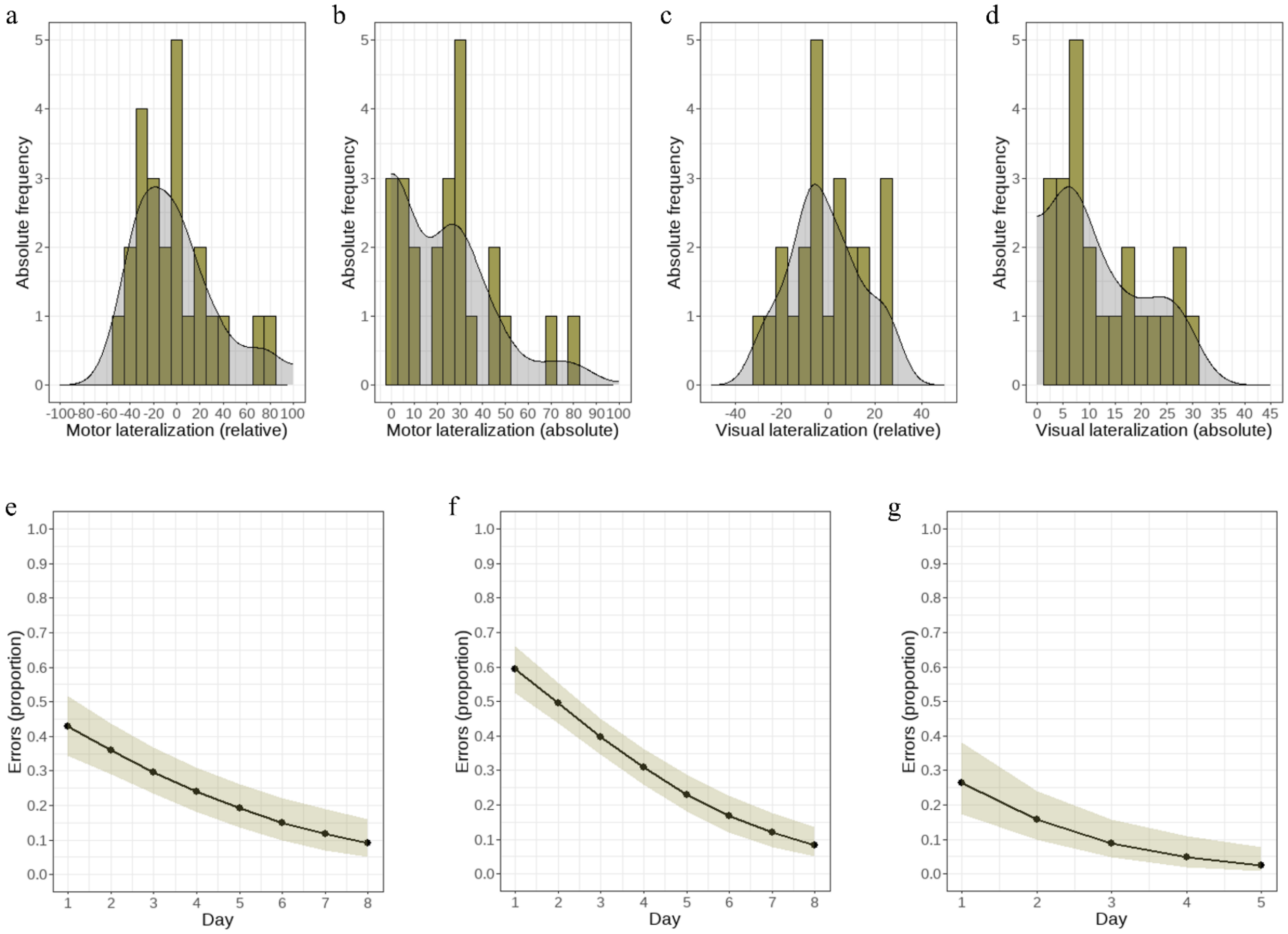

Figure S4. Results of the network analysis performed with alternative variables (the raw data on stress metabolism (operculum-beating frequency), the raw data on cognitive flexibility (number of days to reach criterion in the reversal learning phase), and the data from only the first lateralization trial). Network plot reporting significant associations among traits; each node represents a trait; the color of the nodes represents the domain of the trait (life history = blue; physiology = orange; behavior = light grey; cognition = green); the thickness of an edge (i.e., the line connecting two nodes) represents the strength of the association between nodes; the color of edge represents whether the direction of the association (negative = blue; positive = red).

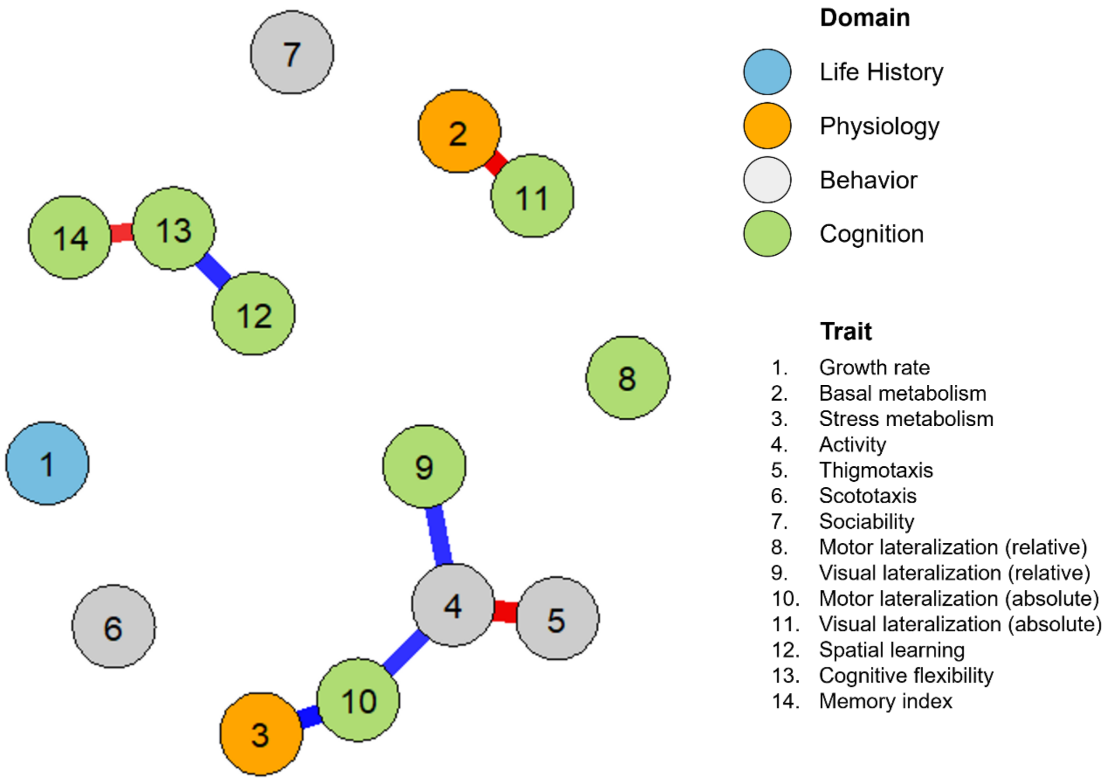

Figure S5. Plots and 95% bootstrapped confidence intervals (CIs) around the estimated edge weights (grey shade). CIs surrounding edge-weights (partial correlation coefficients) of this moderate size imply we should proceed with caution when interpreting our results.

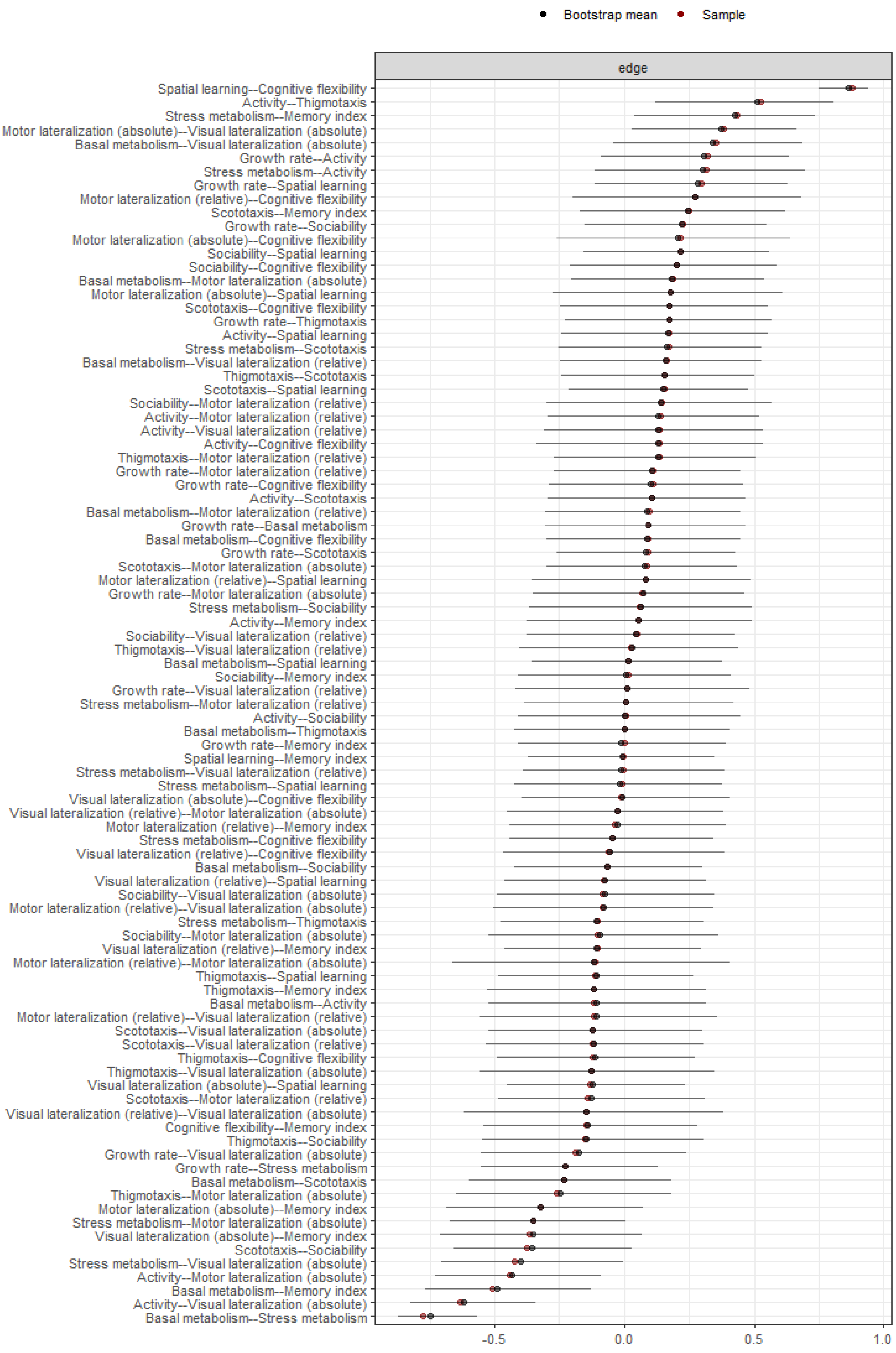

Figure S6. Stability of eigenvector, expected influence and strength, three centrality measures, using case-dropping bootstrapping. CS-coefficient denotes the estimated maximum number of cases that can be dropped from the data to retain, with 95% probability, a correlation of at least 0.7 between statistics based on the original network and statistics computed with less cases.

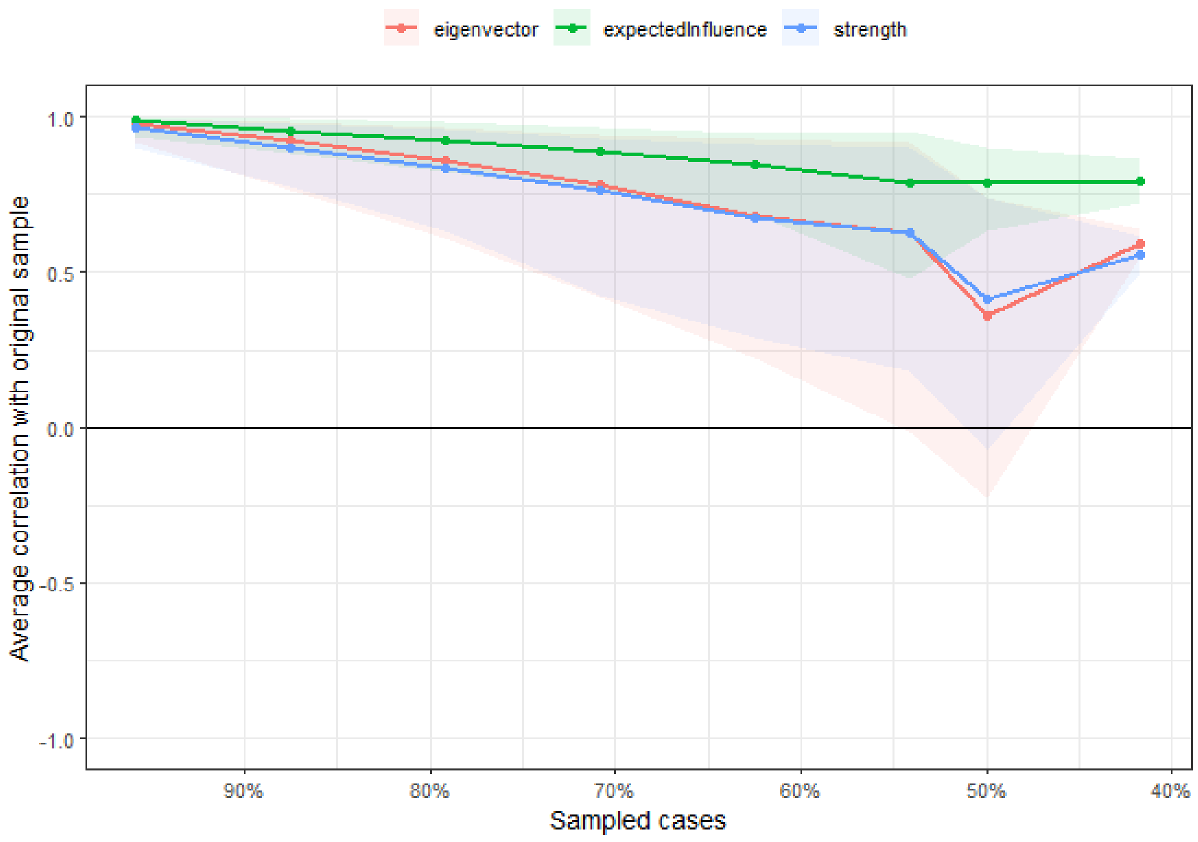

Supplement: arag027_Supplementary_Data [file arag027_supplementary_data.pdf]
